# Identification of Regional Wall Motion Abnormalities by Emergency Physicians in Acute Coronary Syndromes: Systematic Review and Meta-Analysis

**DOI:** 10.5811/westjem.64752

**Published:** 2026-10-18

**Authors:** Eduardo Saadi Neto, Murilo Scapin, Pablo Aparicio Verdun, Francisco Lazaro-Paulina, Danielle Gerberi, Daniel Fiterman Molinari, Ronna L. Campbell, Tobias Kummer

**Affiliations:** * Mayo Clinic, Department of Emergency Medicine, Rochester, Minnesota; † Universidade Estadual De Londrina, Department of Neurosurgery, Londrina, Paraná, Brazil; ‡ Universidad Privada del Valle, Medicine, Department of Health Sciences, Cochabamba, Bolivia; § Hennepin County Medical Center, Department of Emergency Medicine, Minneapolis, Minnesota; || Mayo Clinic Libraries, Rochester, Minnesota; # Mayo Clinic, Department of Emergency Medicine, Jacksonville, Florida

## Abstract

**Introduction:**

Chest pain accounts for approximately six million emergency department (ED) visits annually in the United States. Early detection of acute occlusive myocardial infarction (OMI) is challenging, especially in patients without classic ST-elevation on initial electrocardiography (ECG). Regional wall motion abnormalities (RWMA) often precede ECG changes and can be sensitive indicators of acute coronary occlusion on transthoracic echocardiography (TTE). However, the accuracy of emergency physician-performed TTE for RWMA detection has not been well defined. In this review we aim to determine how accurate emergency physicians are in detecting RWMAs.

**Methods:**

We conducted a systematic review of studies including adult ED patients who underwent cardiac ultrasound performed or interpreted by emergency physicians, with test characteristics compared to cardiology-performed or interpreted echocardiography as the reference standard. Primary outcomes were sensitivity and specificity for RWMA detection. Secondary outcomes included likelihood ratios, and area under the receiver-operator curve (AUC). Risk of bias was assessed using QUADAS-2, and evidence certainty was evaluated using GRADE methodology.

**Results:**

The search identified 2,463 studies, of which 32 underwent full-text review; nine studies including 1,191 participants from the US, Turkey, and Iran are included. Sonographers across studies ranged from medical students and residents to fellowship-trained and senior emergency physicians with varying levels of prior ultrasound and RWMA-specific training. Pooled sensitivity and specificity for RWMA detection were 86% (95% CI, 76–93%) and 85% (95% CI, 70–94%), respectively, with an AUC of 0.918. Pooled positive and negative likelihood ratios were 5.42 (95% CI, 2.51–11.68) and 0.17 (95% CI, 0.09–0.33), respectively. Four of the nine studies were judged to have a high risk of bias in at least one domain. Substantial heterogeneity was observed in the secondary univariate analyses (>80%). The overall certainty of evidence was moderate.

**Conclusion:**

Emergency physician-performed or interpreted cardiac ultrasound demonstrates high accuracy for detecting RWMAs compared with cardiology-performed or interpreted echocardiography. Although heterogeneity was observed across studies, the overall certainty of evidence was moderate. Structured training and standardized protocols may optimize diagnostic performance.

## INTRODUCTION

Chest pain is one of the most common reasons for presentation to the emergency department (ED), accounting for approximately six million visits annually in the United States.^1^ An estimated 600,000 patients present to the ED with acute myocardial infarction annually.^2^ Prompt identification is critical, as cardiovascular disease remains the leading cause of death worldwide, responsible for over 17 million deaths each year.^3^ The electrocardiogram (ECG) is the primary initial diagnostic tool for evaluating patients with suspected acute coronary syndrome and identifying those with acute coronary occlusion, which requires urgent reperfusion therapy. However, in patients without classic ST-elevation (STEMI) on the initial ECG, identifying those who may benefit from urgent coronary intervention remains challenging.^1^

Transthoracic echocardiography (TTE) performed by cardiologists has demonstrated that regional wall motion abnormalities (RWMA) can indicate acute myocardial ischemia and may be detected before diagnostic ECG changes in some patients with acute occlusive myocardial infarction (OMI) and critical coronary stenosis.^4^,^5^ Although comprehensive echocardiography plays a well-established role in evaluating confirmed myocardial infarction, including regional and global cardiac function and mechanical complications, the ability of emergency physicians to reliably detect RWMAs at the bedside using ultrasound remains uncertain.^6^,^7^

Despite this potential, the potential diagnostic accuracy of emergency physician-performed ultrasound for RWMAs currently does not have a formal role in ED, highlighting an important knowledge and literature gap.^1^,^8^ The objective of this systematic review and diagnostic meta-analysis was to evaluate whether emergency physicians can accurately detect RWMAs.

## METHODS

### Study Design

We conducted a diagnostic meta-analysis in accordance with the Preferred Reporting Items for a Systematic Review and Meta-analysis of Diagnostic Test Accuracy Studies (PRISMA-DTA)^9^ and registered in PROSPERO (CRD 420261292335). The review question was framed using a diagnostic accuracy population, intervention, comparison, outcome (PICO) approach.

Participants were adults (18 years of age and older) presenting to the ED with suspected acute coronary syndrome, chest pain, or suspected acute myocardial infarction (including STEMI and non-STEMI [NSTEMI] elevation presentations). The intervention consisted of cardiac ultrasound performed or interpreted by emergency physicians or residents, which included the assessment of RWMAs. We then compared the results via echocardiography performed or interpreted by a cardiologist.

**Population Health Research Capsule**

What do we already know about this issue?

*Regional wall motion abnormalities (RWMAs) on bedside ultrasound may help identify acute coronary occlusion, but their diagnostic accuracy remains uncertain.*

What was the research question?

*How accurately do emergency physicians identify RWMAs using ultrasound?*

What was the major finding of the study?

*Across 9 studies (n = 1,191), pooled sensitivity was 86% and specificity 85% (AUC 0.918).*

How does this improve population health?

*Bedside RWMA assessment is feasible by emergency physicians and may support earlier acute coronary syndrome recognition and risk stratification, complementing standard emergency department evaluation and helping guide timely care.*

The primary outcomes of interest were sensitivity and specificity for the detection of RWMAs. Secondary outcomes were positive and negative likelihood ratios, and the area under the receiver operating characteristic curve (AUC).

### Search Strategy

The literature was searched by a medical librarian for the concepts of RWMA, ultrasonography, the emergency department (ED) setting, and acute coronary syndrome. Search strategies were created using a combination of keywords and standardized index terms. Searches were run on December 16, 2025, in ClinicalTrials.gov, EBSCO Cumulative Index to Nursing and Allied Health Literature, Ovid Cochrane Central Register of Controlled Trials, Ovid Embase, Ovid MEDLINE, Scopus, Web of Science Core Collection, and the World Health Organization’s International Clinical Trials Registry Platform. Results were limited to English, Portuguese and Spanish with conference abstracts and editorials removed. Full search strategies are provided in the appendix.

### Study Selection

Records were managed in Covidence. After removing duplicates, four reviewers (ESN, MS, PAV, FL) independently screened abstracts and full texts; discrepancies were resolved by consensus.

### Eligibility Criteria

We included studies evaluating adult patients presenting to the ED with suspected acute coronary syndrome, chest pain, or acute myocardial infarction, including both STEMI and NSTEMI presentations. To be eligible, studies had to assess cardiac ultrasound examinations performed or interpreted by emergency physicians or emergency medicine trainees. A requirement for inclusion was that the ultrasound examination specifically included an assessment for RWMAs.

Studies were excluded if they involved pediatric populations. We also excluded studies conducted outside the ED setting and those that did not evaluate RWMAs as a clinical outcome. We also excluded case reports, case series, narrative reviews, editorials, and conference abstracts for which the full text was unavailable.

### Outcome Measures

The primary outcomes of interest were the sensitivity and specificity of cardiac ultrasound performed or interpreted by emergency physicians for the detection of RWMAs. Secondary outcomes included positive and negative likelihood ratios and the area under the curve.

### Data Collection Process

After consensus among the authors, the collected data included bibliographic information, study design, setting, sample size, and patient demographics including age, sex, and comorbidities. Intervention details focused on the cardiac views obtained, operator training level, and the technical definition of RWMA. Extracted outcomes included sensitivity, specificity, positive predictive value (PPV), negative predictive value (NPV), raw diagnostic data (true positive [TP], false positive [FP], true negative [TN], and false negative [FN]), and interobserver agreement.

Additional methodological characteristics extracted included the timing of the reference standard relative to the emergency physician-performed ultrasound examination and whether cardiologist interpreters of the reference standard were blinded to the emergency physician ultrasound findings, when reported.

### Risk of Bias and Certainty Assessment

Two reviewers (ESN and PAV) independently assessed the risk of bias for each included study using the Quality Assessment of Diagnostic Accuracy Studies 2 (QUADAS-2) tool. Each study was evaluated across four key domains: patient selection, index test, reference standard, and flow and timing. Any discrepancies between the reviewers during the assessment process were resolved through discussion and consensus. The results of these assessments are reported for all individual studies (Supplemental Material).

To evaluate the overall certainty of the body of evidence, we applied the Grading of Recommendations Assessment, Development, and Evaluation (GRADE) approach. The evidence was graded based on criteria including risk of bias, inconsistency, indirectness, imprecision, and publication bias (Supplemental Material).

### Data Analysis

We conducted all statistical analyses using R (R Foundation for Statistical Computing). When data necessary to build the 2 × 2 table were unavailable, we calculated per the recommendations of the Cochrane handbook.^10^ Pooled sensitivity and specificity were initially estimated using a random effects model with the meta package in R as a univariate approach.

We estimated the primary outcomes (pooled sensitivity and specificity for RWMA detection) using a bivariate random-effects model implemented with the reitsma function from the mada package. This model jointly estimates sensitivity and specificity while accounting for their potential correlation and between-study heterogeneity. Model parameters were estimated using restricted maximum likelihood (REML). Summary sensitivity, FP rate, and their correlation were obtained, and a summary receiver operating characteristic (SROC) curve was generated.

Overall diagnostic performance was quantified using the area under the SROC curve (AUC) and the partial AUC restricted to observed FP rates. Between-study heterogeneity in AUC was assessed using the Zhou and Dendukuri method. We constructed the SROC plots to visualize pooled diagnostic accuracy and study-level variability. Additionally, we calculated study-level likelihood ratios (positive and negative) from the raw 2 × 2 tables. Pooled estimates of positive and negative likelihood ratios were obtained using a random-effects meta-analysis on the log-transformed study-specific ratios.

## RESULTS

We identified 2,463 studies through the search strategy, of which 32 underwent full-text review and nine met inclusion criteria (Figure 1). These studies included a total of 1,191 participants who underwent cardiac ultrasound performed or interpreted by emergency physicians. The included studies consisted of seven prospective observational cohorts and two retrospective observational cohorts. Four studies were conducted in Turkey, three in the US, and two in Iran. Considerable heterogeneity was observed in the training background and experience of sonographers across studies. Participating operators ranged from medical students, residents, and fellowship-trained emergency physicians to senior physicians and attending-level clinicians. Some studies used a single operator, whereas others included multiple sonographers with varying levels of experience. Several studies reported prior focused cardiac ultrasound or point-of-care ultrasound (POCUS) experience, dedicated RWMA training sessions, structured didactic instruction, or supervised hands-on training before study participation. Additional detailed information regarding operator characteristics, training protocols and baseline study characteristics are summarized in Table 1 and 2.

### Secondary Univariate Analysis

As an initial descriptive analysis, pooled sensitivity and specificity were estimated separately using univariate models. Nine studies comprising 467 TP and 77 FN demonstrated a pooled sensitivity of 88% (95% CI, 78–94%) (Figure 2A). The same nine studies, comprising 480 TN and 104 FP, yielded a pooled specificity of 85% (95% CI, 70–94%) (Figure 2B).^1^,^6^,^7^,^11^–^16^

### Primary Bivariate Analysis

Because sensitivity and specificity are correlated diagnostic accuracy measures, the prespecified primary analysis used a bivariate random-effects model that jointly estimates both parameters. Using this model, the pooled sensitivity was 86% (95% CI, 76–93%), with a corresponding pooled specificity of 85% (95% CI, 70–94%). The sROC curve showed excellent diagnostic performance, with an sROC AUC of 0.918 (Figure 3).^1^,^6^,^7^,^11^–^16^

The partial AUC was 0.897, indicating that diagnostic performance remained robust when analysis was restricted to clinically relevant false-positive rates, suggesting that the overall AUC was not driven by unrealistic operating points. We assessed between-study heterogeneity using the Zhou and Dendukuri *I*^2^ statistic, which was 28.4%, indicating low-to-moderate heterogeneity and supporting consistency of diagnostic performance across included studies.

### Likelihood Ratios

Across the included studies, the pooled positive likelihood ratio (LR+) was 5.42 (95% CI, 2.5–11.68), indicating an increase in the probability of disease when the test is positive. The pooled negative likelihood ratio (LR−) was 0.17 (95% CI, 0.09–0.33), reflecting the reduction in the probability of disease when the test is negative.^1^,^6^,^7^,^11^–^16^ (Figure 4)

### Quality assessment

We assessed risk of bias was assessed using the QUADAS-2. Four studies were classified as having a high risk of bias, three as having “some concerns,” and two as having a low risk (Supplemental Figure 1).

The timing of the reference standard and blinding procedures varied across the included studies (Table 3). Most studies performed the reference echocardiographic examination during the index ED encounter or hospitalization, although the reported interval ranged from within 1 hour to later during the same admission when explicitly stated. Blinding of cardiologist interpreters to the emergency physician ultrasound findings was explicitly reported in six studies, whereas three studies did not clearly describe blinding procedures. These factors contributed to the QUADAS-2 assessments for the reference standard and flow and timing domains.

### Grading of recommendations assessment, development, and evaluation assessment

Overall, the certainty of the evidence was determined to be moderate, mainly due to the risk of bias in the included studies; several demonstrated moderate to high risk of bias in the patient selection, flow, and timing domains (Supplemental Table 1).

## DISCUSSION

In this systematic review of nine studies including 1,191 participants, an emergency physician–performed cardiac ultrasound demonstrated excellent diagnostic performance for the detection of RWMAs. The pooled sensitivity and specificity were 86% and 85%, respectively, with an AUC of 0.918, indicating excellent diagnostic performance. The pooled LR+ was 5.42, suggesting that a positive ultrasound substantially increases the probability of RWMAs, while the pooled LR− was 0.17, indicating a strong reduction in the probability of RWMAs when the test is negative. An important finding of this review is that acceptable diagnostic performance was observed despite substantial heterogeneity in operator background, prior ultrasound experience, and RWMA-specific training across included studies. Sonographers ranged from trainees, such as residents to fellowship-trained and senior emergency physicians, with some studies utilizing a single operator and others including multiple clinicians with differing levels of expertise.

Training protocols also varied considerably, ranging from brief didactic sessions and introductory video modules to structured cardiologist-led workshops and supervised hands-on practice (Table 2). Despite this variability, most studies demonstrated favorable agreement with expert interpretation. suggesting that emergency physician assessment of RWMAs may be feasible across a broad range of training environments. The overall results support the potential feasibility and diagnostic performance of emergency physician–performed cardiac ultrasound as a valuable tool for rapid bedside detection of RWMAs in the ED.

In addition to evaluating diagnostic accuracy, several studies compared the time required for emergency physician-performed cardiac ultrasound with the time required for formal echocardiographic assessment. Several studies reported substantial reductions in examination and overall workflow times when ultrasound was performed in the ED by trained physicians. For example, Bracey et al^1^ reported a median ED ultrasound time of 10 minutes (interquartile range [IQR] 3–6) compared with to median consultative cardiology echocardiography time of 952 minutes (IQR 59–1,356). Similarly, Karanci et al^13^ observed a median ED ultrasound time of 20 minutes (IQR 15–25) versus cardiology-performed echocardiography at 120 minutes (IQR 120–140), while Croft et al^6^ reported median times of 810 minutes (IQR 535–1,410) for ED ultrasound compared with 1,380 minutes (IQR 540–2,280) for standard ultrasound.

The expanding role of POCUS has transformed emergency medicine, becoming an established component of bedside evaluation for trauma, respiratory disease, abdominal aortic aneurysm, early pregnancy complications, and hemodynamic assessment.^17^–^21^ Within this broader context, these findings suggest that emergency physician–performed cardiac ultrasound may permit earlier bedside assessment for RWMAs than formal echocardiography in some clinical settings. In patients with suspected OMI, particularly when ECG findings are nondiagnostic or equivocal, early identification of RWMAs may provide complementary diagnostic information that supports earlier recognition of ischemia, facilitates expedited cardiology consultation, and may ultimately shorten time to reperfusion when interpreted alongside the clinical presentation and ECG findings.

However, the included studies primarily evaluated examination timing and diagnostic agreement rather than downstream clinical outcomes, such as time to reperfusion, door-to-balloon time, missed OMI, changes in treatment, or patient outcomes. Therefore, these results should not be interpreted as demonstrating that earlier RWMA detection leads to earlier treatment or improved clinical outcomes. Prospective studies specifically designed to evaluate these outcomes are needed.

The findings of this review also highlight the importance of structured training and standardized ultrasound education to optimize the diagnostic performance of emergency physician-performed ultrasound. Variability in operator experience from residents to attending physicians likely contributed to some of the differences observed across studies, underscoring the need for formalized curricula that include both didactic knowledge and hands-on scanning under supervision.^22^ Standardization efforts, such as the development of core POCUS training frameworks and certification pathways, seek to ensure that emergency physicians achieve consistent competency across essential applications, including cardiac assessment.^23^ Comprehensive residency-based POCUS training programs have been shown to increase use and proficiency among emergency trainees, supporting the notion that structured education enhances both skill acquisition and clinical integration of ultrasound.^24^ Additionally, longitudinal faculty development and certification initiatives can improve skill retention, confidence, and teaching capability, helping reduce inter-operator variability and promote sustained competency within EDs.^25^

A previous systematic review and meta-analysis by Albaroudi et al^26^ demonstrated substantial agreement between emergency physician sonographers and expert interpretation for visual estimation of left ventricular systolic function, with pooled sensitivity and specificity of 89% and 85%, respectively. In contrast, the present review specifically evaluated the detection of RWMAs, a more nuanced echocardiographic finding with important implications for the identification of acute coronary syndromes and other cardiac pathologies. Despite differences in study populations, operator experience, and training protocols, our pooled estimates demonstrated similarly favorable diagnostic performance, suggesting that emergency physicians may be capable not only of identifying gross abnormalities in global systolic function, but also of recognizing more localized myocardial dysfunction using point-of-care echocardiography.

In summary, this systematic review provides the first comprehensive synthesis of evidence demonstrating that emergency physician-performed cardiac ultrasound has high diagnostic accuracy for detecting RWMAs. To our knowledge, no prior systematic review has specifically evaluated the diagnostic performance of bedside cardiac ultrasound for RWMAs performed by emergency physicians, an important gap this meta-analysis addresses. By quantifying pooled sensitivity, specificity, and likelihood ratios across multiple studies, our findings support the potential feasibility and diagnostic accuracy of emergency physician-performed cardiac ultrasound for RWMA detection. This work lays the foundation for future investigations into patient-centered outcomes, standardization of training, and the real-world implementation of emergency physician–performed cardiac ultrasound.

## LIMITATIONS

Several limitations of this meta-analysis should be acknowledged. First, the included studies were heterogeneous in terms of study design, operator expertise, and patient selection. Seven of the nine studies were prospective observational cohorts, and two were retrospective, which may have introduced variability in patient enrollment, timing of the ultrasound examination, and outcome assessment. Patient selection criteria also differed (eg, some studies excluded patients with STEMI). These differences may have introduced selection and spectrum bias. Additionally, operators ranged from emergency medicine residents to attending physicians with varying levels of ultrasound experience, potentially influencing the diagnostic performance reported in individual studies.

Additionally, the prevalence of RWMAs also varied substantially across the included studies, ranging from 8% to 72%, with an average prevalence of approximately 45%. This average is considerably higher than would be expected among a general population of patients presenting to the ED with undifferentiated chest pain and may limit the generalizability of these findings to lower-prevalence clinical settings. Because PPVs and NPVs are highly dependent on disease prevalence, the wide variation in RWMA prevalence across the included studies also limits the validity and generalizability of pooled PPV and NPV estimates; therefore, these measures were ultimately not pooled or reported.

Second, univariate analyses of sensitivity and specificity demonstrated higher heterogeneity compared to the bivariate model. This discrepancy is likely attributable to the univariate approach, which treats sensitivity and specificity independently without accounting for their inherent negative correlation. In contrast, the bivariate random effects model jointly estimates sensitivity and specificity and incorporates the correlation between these measures, resulting in lower observed heterogeneity. Therefore, the bivariate model provides a more reliable estimate of overall diagnostic performance.

Third, the risk of bias was moderate to high in several studies, particularly in the patient selection and flow and timing domains. Differences in RWMA definitions, scanning protocols, image acquisition, operator training, and reference interpretation may also limit the comparability and generalizability of the results. Publication bias was not formally assessed with a funnel plot because only 9 studies were included; current methodological guidance cautions against interpreting funnel plots when fewer than 10 studies are available because of limited statistical power and potentially misleading visual asymmetry. Additionally, although the Reitsma bivariate model implemented in the mada package provides an AUC estimate, it does not generate an analytical 95% CI. A simulation-based approach was explored with statistical consultation, but the resulting interval was not sufficiently stable or reliable; therefore, the AUC is reported as a point estimate only to avoid overstating its precision.

Despite these limitations, the pooled estimates demonstrated robust diagnostic accuracy for emergency physician-performed cardiac ultrasound in detecting RWMAs, supported by consistent results in sensitivity, specificity, and SROC analyses.

## CONCLUSION

Cardiac ultrasound performed or interpreted by emergency physicians showed high diagnostic accuracy for detecting RWMAs, with favorable sensitivity, specificity, and likelihood ratios. Despite moderate heterogeneity across studies, the overall certainty of the evidence was moderate, supporting the potential feasibility of cardiac ultrasound as a reliable bedside assessment tool for RWMAs. Although these results are promising, further research is needed to reduce bias through well-designed prospective diagnostic studies using standardized training protocols and rigorous methodology. Structured training and standardized protocols may further optimize diagnostic performance. This meta-analysis fills an important gap in the literature, providing a quantitative summary of the available evidence to guide implementation and future research.

## Supplementary Information







## Figures and Tables

**Figure 1:**
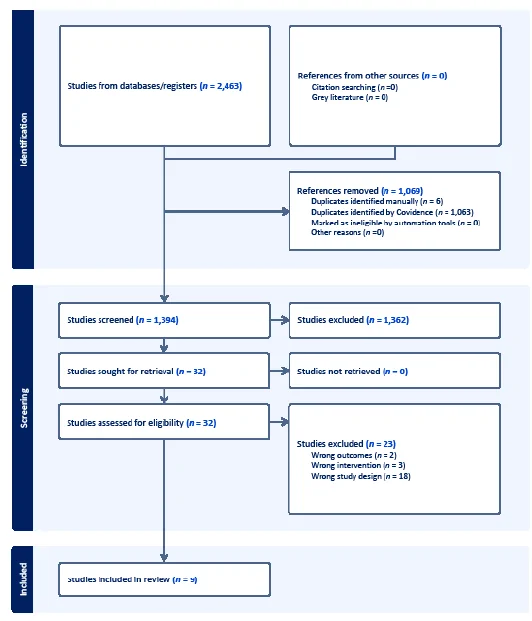
PRISMA flowchart for included studies of identification of regional wall motion abnormalities by emergency physicians. *PRISMA*, Preferred Reporting Items for Systematic Reviews and Meta-Analyses.

**Figure 2:**
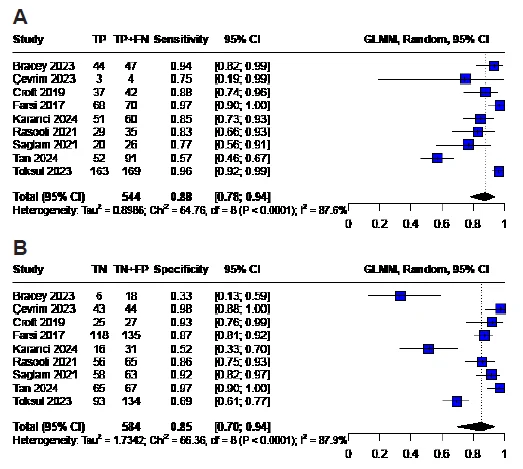
Forest plot for sensitivity (A) and specificity (B) of “emergency physician-performed ultrasound to detect regional wall motion abnormalities” (Univariate analysis) *FN*, false negative; *FP*, false positive; *GLMM*, generalized linear mixed model; *TP*, true positive.

**Figure 3:**
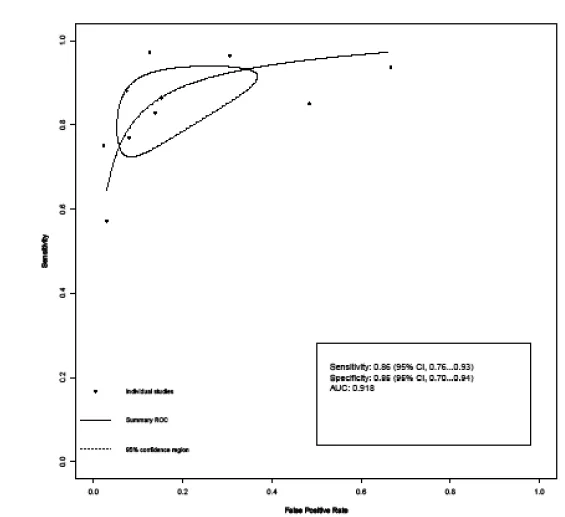
Summary receiver operating characteristic of emergency physician-performed ultrasound to detect regional wall motion abnormalities. *EM*, emergency medicine; *ROC*, receiver operating characteristic; *RMWA*, regional wall motion abnormality.

**Figure 4:**
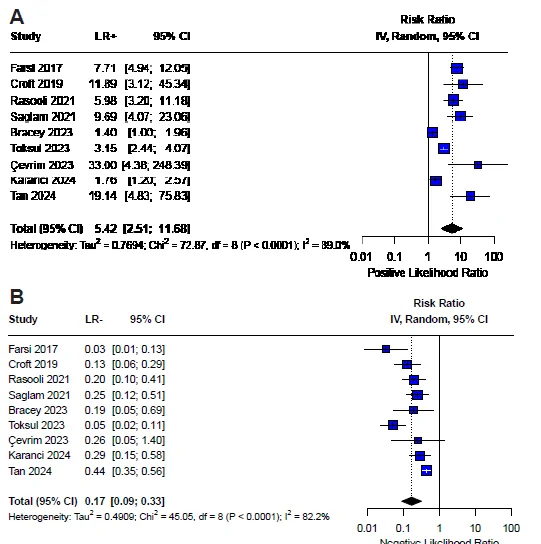
Forest plots for positive (**A**) and negative (**B**) likelihood ratios of emergency physician-performed ultrasound to detect regional wall motion abnormalities. *IV*, inverse variance; *LR*, likelihood ratio.

**Table 1:** Study baseline characteristics for included studies of “Identification of regional wall motion abnormalities by emergency physicians.”

Study	Country	Sample size	Age, years (mean [SD])	Female (n, %)	Comorbidities	Inclusion criteria	Exclusion criteria	Cardiac views assessed on ED-performed US	RWMA definition
Farsi 2017	Iran	205	61 (17)	102 (49.8%)	N/A	Adults (≥ 18 years of age) presenting to the ED with suspected cardiac emergency.	US would interfere with treatment.	apical 4-chamber; parasternal long-axis; parasternal short-axis; subxiphoid 4-chamber.	N/A
Croft 2019	USA	69	62.0 (12.47)	20 (29.0%)	Known CAD^*^: 16 (27%) Hypertension: 42 (76%) Diabetes mellitus: 10 (18%)	Adults (≥ 18 years of age) presenting to the ED with ECG-confirmed STEMI.	No exclusion criteria.	apical 4-chamber; parasternal long-axis; parasternal short-axis.	N/A
Rasooli 2021	Iran	100	54.1 ± 11.5	35 (35.0%)	Hypertension: 49 (49%) Diabetes mellitus: 19 (19.0%) Dyslipidemia: 33 (33.0%)	Adults (≥ 18 years of age) presenting to the ED with acute coronary syndrome (ACS).	STEMI needing primary PCI; pacemaker implantation.	apical 2-chamber; apical 4-chamber; long-axis; short-axis.	Akinesia, dyskinesia, or hypokinesia of the left ventricle.
Saglam 2021	Turkey	89	59.6 (14.04)	25 (28.1%)	Hypertension: 45 (50.6%) Diabetes mellitus: 18 (20.2%) Dyslipidemia: 21/(23.6%)	Adults (≥ 18 years of age) presenting to the ED with chest pain, HEART score ≥ 4, and a likely ACS diagnosis.	STEMI, chronic heart failure, tachycardia, or bradycardia.	apical 2-chamber; apical 4-chamber; parasternal long-axis; parasternal short-axis.	Segmental left ventricular akinesia, dyskinesia, or hypokinesia.
Bracey 2023	USA	65	62 (14)	23 (35.4%)	Known CAD^*^: 19 (29%) Hypertension: 47 (72%) Diabetes mellitus: 20 (31%) Dyslipidemia: 37 (57%)	Patients > 17 years of age presenting to the ED with high suspicion for ACS.	If no formal echocardiography for comparison	apical 2-chamber; apical 4-chamber; parasternal long; parasternal short-axis.	≥ 1 Segmental left ventricular wall abnormality.
Toksul 2023	Turkey	303	63.14 (0.89)	106 (35.0%)	Known CAD^*^: 102 (33.7%) Hypertension: 62 (20.5%) Diabetes mellitus: 22 (7.3%)	Adults (≥ 18 years of age) presenting to the ED with chest pain and shortness of breath.	No exclusion criteria.	apical 2-chamber; apical 4-chamber; parasternal long; parasternal short-axis.	Segmental left ventricular akinesia, dyskinesia, or hypokinesia.
Çevrim 2023	Turkey	48	49 (13)	24 (50.0%)	N/A	Adults (≥ 18 years of age) presenting to the ED with chest pain.	Prior CAD; CABG/stent; ST-elevation on admission ECG; low EF needing resuscitation on admission.	apical 2-chamber; apical 4-chamber; parasternal long-axis; parasternal short-axis.	SWMA score > 1.1.
Karanci 2024	Turkey	91	58.5 (8.5)	29 (31.9%)	Known CAD^*^: 44 (48%) Hypertension: 39 (43%) Diabetes mellitus: 23 (25%) Dyslipidemia: 24 (26%)	Adults (≥ 18 years of age) presenting to the ED with suspected ACS.	ST-elevation; no PCI within 72 hrs; no records; US not feasible; unsuitable conditions for US.	apical 2-chamber; apical 4-chamber; parasternal long; parasternal short-axis.	N/A
Tan 2024	USA	221	64.8 (13.4)	75 (33.9%)	N/A	Adults (≥ 18 years of age) presenting to the ED with suspected ACS who underwent diagnostic cardiac catheterization within 72 hours of ED presentation.	ED ECG met cath-lab activation criteria or inadequate images for RWMA.	N/A	Interpreted qualitatively as present, absent, or uninterpretable.

*ACS*, acute coronary syndrome; *CABG*, coronary artery bypass graft; *CAD*, coronary artery disease; *ECG*, electrocardiogram; *ED*, emergency department; *EF*, ejection fraction; *N/A*, not available; *PCI*, percutaneous coronary intervention; *RWMA*, regional wall motion abnormality; *STEMI*, ST-elevation myocardial infarction; *SWMA*, segmental wall movement abnormality; *US*, ultrasound.

**Table 2:** Emergency physician operator training and diagnostic accuracy across included studies of “identification of regional wall motion abnormalities by emergency physicians.”

Study	EM US operator training	US Sensitivity, % (95% CI)	US Specificity, % (95% CI)	PPV/NPV, % (95% CI)
Farsi 2017	EM residents (*n* = 17): attended an ASE-protocol US workshop (two 2-h theory + three 2-h practical sessions).	97 (89–99)	87 (80–92)	69 (48–85)/98 (95–99)
Croft 2019	EM residents (*n* = 9; 5 PGY-1; 3 PGY-2; 1 PGY-3): had prior POCUS experience (≥ 25 TTE; 150 total POCUS studies), had no prior RWMA training, and completed two introductory video modules.	88% (75–96)	92% (75–99)	95 (83–99)/83 (68–92)
Rasooli 2021	EM resident (PGY-3): received 2 h of didactics plus 20 h of supervised hands-on practice under two cardiologists (ACEP-guided).	82.9 (66.4–93.4)	86.2 (75.3–93.5)	76.3 (59.8–88.6)/90.3 (80.1–96.4)
Saglam 2021	Emergency physicians (*n* = 3): had 5 years of bedside US experience, completed an accredited POCUS course, and then received an additional 6-h cardiologist-led RWMA training session (3-h didactic + 3-h hands-on).	76.9 (56.4–91.0)	92.1 (82.4–97.4)	80.0 (62.7–90.5)/90.6 (82.7–95.1)
Bracey 2023	EM resident/fellow/attending: all received a 30-min RWMA-identification presentation before performing study US.	94 (82–98)	35 (15–61)	80 (67–89)/67 (31–91)
Toksul 2023	Emergency physicians: had attended at least one US training course.	96.4	69.4	79.9/93.9
Çevrim 2023	Emergency physicians + assistant: had >2 years of experience; the physician had completed a certified basic emergency US course plus targeted emergency echocardiography training.	75 (6–79)	97 (83–100)	75 (9–90)/97 (80–99)
Karanci 2024	Emergency physicians: had ≥2 years of US experience and held basic and advanced US certificates.	85 (73–92)	53 (36–69)	77 (65–85)/65 (46–81)
Tan 2024	Fellowship-trained emergency physicians (*n* = 3), emergency residents (*n* = 2), and medical student (*n* = 1): all attending/resident investigators had received emergency ultrasound training during residency and/or fellowship.	57 (47–67)	96 (87–100)	N/A/N/A

*ASE*, American Society of Echocardiography; *ED*, emergency department; *EF*, ejection fraction; *EM*, emergency medicine; *N/A*, not available; *NPV*, negative predictive value; *PCI*, percutaneous coronary intervention; *PPV*, positive predictive value; *TTE*, transthoracic echocardiography; *US*, ultrasound.

**Table 3:** Reference standard characteristics and flow/timing assessment of included studies of “identification of regional wall motion abnormalities by emergency physicians”

Study	Reference standard timing	Cardiologist blinded?	Flow & Timing considerations
Farsi 2017	Reference US performed as soon as possible after EM US (exact interval not reported).	Yes	All included patients underwent both examinations; no post-enrollment exclusions.
Croft 2019	Reference US performed within 24 hrs after reperfusion and as close as possible to the study ultrasound.	Yes	6 patients excluded before analysis because of withdrawal, incomplete data, or inadequate images.
Rasooli 2021	Reference US performed within 1 hr of EM examination.	Yes	All enrolled patients underwent both examinations with no post-enrollment exclusions.
Saglam 2021	Reference US during the same ED encounter (exact interval not reported).	NR	22 patients excluded before analysis (17 declined participation; 5 inadequate image quality).
Bracey 2023	Reference US during the index admission	NR	11 patients excluded because no reference US was available.
Toksul 2023	Reference evaluation performed during the same clinical encounter (exact interval not reported).	Yes	All enrolled patients underwent both assessments.
Çevrim 2023	Both US performed during the same ED encounter before completion of the 6-hr ACS observation protocol.	Yes	14 patients excluded before analysis (5 withdrew; 9 inadequate image quality).
Karancı 2024	Median door-to-FOCUS: 20 mins Median door-to-TTE: 120 mins	NR	All enrolled patients underwent both examinations and were included in the final analysis.
Tan 2024	Reference US performed during the same admission (exact interval not reported).	NR	Reference US available in 158/221 patients, introducing potential verification bias.

*ED*, emergency department; *EM*, emergency medicine; *NR*, not reported; *US*, ultrasound.
