# Supplementary material for: Identification of Regional Wall Motion Abnormalities by Emergency Physicians in Acute Coronary Syndromes: Systematic Review and Meta-Analysis
Source: West J Emerg Med. 2026 Oct 18;27(6):1703–13. doi: 10.5811/westjem.64752 (PMC13761796; doi:10.5811/westjem.64752)
Supplement: Supplementary file 1 [file wjem-27-1703-s001.docx]

**Identification of Regional Wall Motion Abnormalities by Emergency Medicine Physicians: A systematic Review and Diagnostic Meta-analysis**

***Search strategy***

| ***Databases & Registers*** | ***# of initial results*** |
| --- | --- |
| ***Central*** | ***17*** |
| ***CINAHL*** | ***151*** |
| ***ClinicalTrials.gov*** | ***2*** |
| ***Embase*** | ***641*** |
| ***ICTRP*** | ***2*** |
| ***Medline*** | ***461*** |
| ***Scopus*** | ***845*** |
| ***Web of Science*** | ***344*** |
| ***Totals*** | ***2463*** |

***CINAHL with Full Text via EBSCO (1963+):***

| ***1*** | ***XB(wall-motion or RWMA* or hypokine* or akine* or dyskine* or tardodiskene* or paradoxical or distort*)*** |
| --- | --- |
| ***2*** | ***MH "Ultrasonography+" OR XB(echo* or ultraso* or sonogra* or POCUS or ((real-time or POC* or point-of-care or handheld or hand-held or bedside or bed-side or onsite or on-site) N1 US))*** |
| ***3*** | ***MH "Emergency Service+" OR MH "Emergency Patients" OR MH "Emergency Room Visits" OR MH "Emergency Medicine" OR XB((emergency or A-and-E or A-E or A&E or A-&-E) N2 (department* or medicine or room* or unit* or ward*)) OR SO(Emergency)*** |
| ***4*** | ***MH "Acute Coronary Syndrome" OR MH "Myocardial Infarction+" OR MH "Chest Pain+" OR XB(cardi* or coronary or myocardi* or ACS or MI or infarct* or stemi or minoca or ((chest or thora*) N2 (pain* or discomfort)) or texidor* or precordial-catch or stenocardia* or angina or pectoris or thoracalgi* or thoracodyni* or heart-attack*))*** |
| ***5*** | ***1 AND 2 AND 3 AND 4*** |
| ***6*** | ***( ( (MH "Child+") OR (MH "Adolescence+") ) NOT (MH "Adult+") )*** |
| ***7*** | ***5 not 6*** |

***ClinicalTrials.gov – (2000+):***

***("wall motion" OR rwma) AND (sonography OR ultrasound OR POCUS OR echo OR echography OR echocardiogram) AND emergency***

***Cochrane Central Register of Controlled Trials (CCTR) via Ovid (1991+)):***

| ***#*** | ***Query*** | ***Results from 16 Dec 2025*** |
| --- | --- | --- |
| ***1*** | ***(wall-motion or RWMA* or hypokine* or akine* or dyskine* or tardodiskene* or paradoxical or distort*).ab,hw,ti.*** | ***8,818*** |
| ***2*** | ***(echo* or ultraso* or sonogra* or POCUS or ((real-time or POC* or point-of-care or handheld or hand-held or bedside or bed-side or onsite or on-site) adj1 US)).ab,hw,ti.*** | ***91,613*** |
| ***3*** | ***(((emergency or A-and-E or A-E or AE or A&E or A-&-E) adj2 (department* or medicine or room* or unit* or ward*)) or urgent-care or emergicent*).ab,ti,hw,jw. or (ED or ER).ti.*** | ***25,417*** |
| ***4*** | ***(cardi* or coronary or myocardi* or ACS or MI or infarct* or stemi or minoca or ((chest or thora*) adj2 (pain* or discomfort)) or texidor* or precordial-catch or stenocardia* or angina or pectoris or thoracalgi* or thoracodyni* or heart-attack*).ab,hw,ti.*** | ***264,381*** |
| ***5*** | ***1 and 2 and 3 and 4*** | ***17*** |

***Embase via Ovid (1974+):***

| ***#*** | ***Query*** | ***Results from 16 Dec 2025*** |
| --- | --- | --- |
| ***1*** | ***heart left ventricle wall motion/ or (wall-motion or RWMA* or hypokine* or akine* or dyskine* or tardodiskene* or paradoxical or distort*).ab,kf,ti.*** | ***214,427*** |
| ***2*** | ***echography/ or exp echocardiography/ or ultrasound/ or (echo* or ultraso* or sonogra* or POCUS or ((real-time or POC* or point-of-care or handheld or hand-held or bedside or bed-side or onsite or on-site) adj1 US)).ab,kf,ti.*** | ***1,687,364*** |
| ***3*** | ***emergency ward/ or hospital emergency service/ or emergency physician/ or emergency medicine/ or (((emergency or A-and-E or A-E or AE or A&E or A-&-E) adj2 (department* or medicine or room* or unit* or ward*)) or urgent-care or emergicent*).ab,kf,ti,dq,ox,hw,jx. or (ED or ER).ti.*** | ***501,504*** |
| ***4*** | ***exp acute coronary syndrome/ or exp heart infarction/ or exp thorax pain/ or (cardi* or coronary or myocardi* or ACS or MI or infarct* or stemi or minoca or ((chest or thora*) adj2 (pain* or discomfort)) or texidor* or precordial-catch or stenocardia* or angina or pectoris or thoracalgi* or thoracodyni* or heart-attack*).ab,kf,ti.*** | ***3,395,069*** |
| ***5*** | ***1 and 2 and 3 and 4*** | ***2,410*** |
| ***6*** | ***limit 5 to (english or portuguese or spanish)*** | ***2,375*** |
| ***7*** | ***exp juvenile/ not exp adult/*** | ***2,864,387*** |
| ***8*** | ***6 not 7*** | ***2,319*** |
| ***9*** | ***limit 8 to (books or chapter or conference abstract or "conference review" or editorial)*** | ***1,678*** |
| ***10*** | ***8 not 9*** | ***641*** |

***International Clinical Trials Registry Platform (ICTRP) from the World Health Organization (2005+) – *standard interface*:***

***((wall AND motion) OR RWMA) AND (sonograph* OR ultraso* OR POCUS OR echo*) AND emergency***

***MEDLINE via Ovid (1946+ and Epub Ahead of Print, In-Process & Other Non-Indexed Citations and Ovid MEDLINE(R) Daily):***

| ***#*** | ***Query*** |
| --- | --- |
| ***1*** | ***(wall-motion or RWMA* or hypokine* or akine* or dyskine* or tardodiskene* or paradoxical or distort*).ab,kf,ti.*** |
| ***2*** | ***exp Ultrasonography/ or (echo* or ultraso* or sonogra* or POCUS or ((real-time or POC* or point-of-care or handheld or hand-held or bedside or bed-side or onsite or on-site) adj1 US)).ab,kf,ti.*** |
| ***3*** | ***Emergency Service, Hospital/ or Emergency Medicine/ or (((emergency or A-and-E or A-E or AE or A&E or A-&-E) adj2 (department* or medicine or room* or unit* or ward*)) or urgent-care or emergicent*).ab,kf,ti,hw,jw. or (ED or ER).ti.*** |
| ***4*** | ***Acute Coronary Syndrome/ or exp Myocardial Infarction/ or exp Chest Pain/ or (cardi* or coronary or myocardi* or ACS or MI or infarct* or stemi or minoca or ((chest or thora*) adj2 (pain* or discomfort)) or texidor* or precordial-catch or stenocardia* or angina or pectoris or thoracalgi* or thoracodyni* or heart-attack*).ab,kf,ti.*** |
| ***5*** | ***1 and 2 and 3 and 4*** |
| ***6*** | ***limit 5 to (english or portuguese or spanish)*** |
| ***7*** | ***(exp infant/ or exp child/ or adolescent/) not exp adult/*** |
| ***8*** | ***6 not 7*** |
| ***9*** | ***limit 8 to editorial*** |
| ***10*** | ***8 not 9*** |

***Scopus via Elsevier (1788+):***

***( ( TITLE-ABS-KEY ( wall-motion OR RWMA* OR hypokine* OR akine* OR dyskine* OR tardodiskene* OR paradoxical OR distort* ) ) AND ( TITLE-ABS-KEY ( ( echo* OR ultraso* OR sonogra* OR POCUS OR ( ( real-time OR POC* OR point-of-care OR handheld OR hand-held OR bedside OR bed-side OR onsite OR on-site ) W/1 US ) ) ) ) AND ( ( TITLE-ABS-KEY ( ( ( ( emergency OR A-and-E OR A-E OR AE OR A&E OR A-&-E ) W/2 ( department* OR medicine OR room* OR unit* OR ward* ) ) OR urgent-care OR emergicent* ) ) OR SRCTITLE ( emergency ) ) ) AND ( TITLE-ABS-KEY ( ( cardi* OR coronary OR myocardi* OR ACS OR MI OR infarct* OR stemi OR minoca OR ( ( chest OR thora* ) W/2 ( pain* OR discomfort ) ) OR texidor* OR precordial-catch OR stenocardia* OR angina OR pectoris OR thoracalgi* OR thoracodyni* OR heart-attack* ) ) ) ) AND NOT ( ( TITLE ( p*ediatric* OR infan* OR newborn* OR neonat* OR toddler* OR child* OR adolesc* OR teen* OR youth ) OR SRCTITLE ( p*ediatric* OR infan* OR newborn* OR neonat* OR toddler* OR child* OR adolesc* OR teen* OR youth ) ) ) AND ( EXCLUDE ( DOCTYPE , "ch" ) OR EXCLUDE ( DOCTYPE , "ed" ) ) AND ( LIMIT-TO ( LANGUAGE , "English" ) OR LIMIT-TO ( LANGUAGE , "Portuguese" ) OR LIMIT-TO ( LANGUAGE , "Spanish" ) ) AND ( LIMIT-TO ( EXACTKEYWORD , "Emergency Ward" ) )***

***Web of Science Core Collection via Clarivate Analytics (Science Citation Index Expanded 1975+ & Emerging Sources Citation Index 2015+):***

| ***#7*** | ***#5 NOT #6*** |
| --- | --- |
| ***#6*** | ***(TI=(p$ediatric* or infan* or newborn* or neonat* or toddler* or child* or adolesc* or teen* or youth )) OR SO=(p$ediatric* or infan* or newborn* or neonat* or toddler* or child* or adolesc* or teen* or youth)*** |
| ***#5*** | ***#1 AND #2 AND #3 AND #4 and English or Spanish or Portuguese (Languages) and Editorial Material or Meeting Abstract (Exclude – Document Types)*** |
| ***#4*** | ***TS=(cardi* or coronary or myocardi* or ACS or MI or infarct* or stemi or minoca or ((chest or thora*) NEAR/2 (pain* or discomfort)) or texidor* or precordial-catch or stenocardia* or angina or pectoris or thoracalgi* or thoracodyni* or heart-attack*)*** |
| ***#3*** | ***(TS=(((emergency or A-and-E or A-E or A&E or A-&-E) NEAR/2 (department* or medicine or room* or unit* or ward*)))) OR SO=(Emergency)*** |
| ***#2*** | ***(echo* or ultraso* or sonogra* or POCUS or ((real-time or POC* or point-of-care or handheld or hand-held or bedside or bed-side or onsite or on-site) NEAR/1 US)) (Topic)*** |
| ***#1*** | ***wall-motion or RWMA* or hypokine* or akine* or dyskine* or tardodiskene* or paradoxical or distort* (Topic)*** |
