# Supplementary material for: Identification of Regional Wall Motion Abnormalities by Emergency Physicians in Acute Coronary Syndromes: Systematic Review and Meta-Analysis
Source: West J Emerg Med. 2026 Oct 18;27(6):1703–13. doi: 10.5811/westjem.64752 (PMC13761796; doi:10.5811/westjem.64752)
Supplement: Supplementary file 2 [file wjem-27-1703-s002.docx]

***Supplemental Figure 1. Risk of bias* of “identification of regional wall motion abnormalities by emergency physicians”**
