# Supplementary material for: Identification of Regional Wall Motion Abnormalities by Emergency Physicians in Acute Coronary Syndromes: Systematic Review and Meta-Analysis
Source: West J Emerg Med. 2026 Oct 18;27(6):1703–13. doi: 10.5811/westjem.64752 (PMC13761796; doi:10.5811/westjem.64752)
Supplement: Supplementary file 3 [file wjem-27-1703-s003.docx]

***Supplemental Table 1. Grading of recommendations assessment, development, and evaluation of “*identification of regional wall motion abnormalities by emergency physicians*”***

**Question**: Should Emergency Physician Ultrasound be used to diagnose RWMA in patients under suspicion for Myocardial infarction?

| Sensitivity | 0.86 (95% CI, 0.76 to 0.93) |
| --- | --- |
| Specificity | 0.85 (95% CI, 0.70 to 0.92) |

| Prevalences | 45% |
| --- | --- |

| Outcome | № of studies (№ of patients) | Study design | Factors that may decrease certainty of evidence | | | | |  |  |
| --- | --- | --- | --- | --- | --- | --- | --- | --- | --- |
|  |  |  | Risk of bias | Indirectness | Inconsistency | Imprecision | Publication bias | pre-test probability of 45% | Test accuracy CoE |
| **True positives** (patients with RWMA) | 9 studies 1191 patients | cross-sectional, retrospective and prospective observational studies | serious^a^ | not serious | not serious^b^ | not serious | none | 387 (342 to 419) | ⨁⨁⨁◯ Moderate^a,b^ |
| **False negatives** (patients incorrectly classified as not having RWMA) |  |  |  |  |  |  |  | 63 (31 to 108) |  |
| **True negatives** (patients without RWMA) | 9 studies 1191 patients | cross-sectional, retrospective and prospective observational studies | serious^a^ | not serious | not serious^b^ | not serious | none | 468 (385 to 506) | ⨁⨁⨁◯ Moderate^a,b^ |
| **False positives** (patients incorrectly classified as having RWMA) |  |  |  |  |  |  |  | 82 (44 to 165) |  |

**Explanations**

a. Several studies had moderate to high risk of bias in patient selection and flow/timing domains.

b. Observed heterogeneity is likely attributable to expected clinical and methodological differences across studies, including operator experience, patient spectrum, and study design, rather than true inconsistency in test performance.
